# Biomarkers of inflammation and colorectal cancer risk

**DOI:** 10.3389/fonc.2025.1514009

**Published:** 2025-02-06

**Authors:** Yuting Li, Yuexin Luo, Yue Ran, Furong Lu, You Qin

**Affiliations:** ^1^ Cancer Center, Union Hospital, Tongji Medical College, Huazhong University of Science and Technology, Wuhan, China; ^2^ Institute of Radiation Oncology, Union Hospital, Tongji Medical College, Huazhong University of Science and Technology, Wuhan, China; ^3^ Hubei Key Laboratory of Precision Radiation Oncology, Wuhan, China; ^4^ First Clinic School, Union Hospital, Tongji Medical College, Huazhong University of Science and Technology, Wuhan, China; ^5^ Second Clinic School, Tongji Hospital, Tongji Medical College, Huazhong University of Science and Technology, Wuhan, China; ^6^ Department of Integrated Traditional Chinese and Western Medicine, Union Hospital, Tongji Medical College, Huazhong University of Science and Technology, Wuhan, China

**Keywords:** colorectal cancer, inflammation, C-reactive protein (CRP), interleukin (IL)-6, tumor necrosis factor-alpha (TNF-α)

## Abstract

Globally, colorectal malignancy ranks among the most prevalent forms of cancer and stands as the third principal cause of cancer-associated mortality. Recent studies indicate that inflammatory processes play a significant role in the initiation and advancement of various malignancies, colorectal cancer included. It explores inflammatory biomarkers, with C-reactive protein (CRP) being a key focus. While CRP’s elevation during inflammation is linked to tumorigenesis, studies on its association with CRC risk are inconsistent, showing gender and methodological differences. Interleukin-6 (IL-6), TNF - α, and their receptors also play roles in CRC development, yet research findings vary. Adiponectin and leptin, secreted by adipocytes, have complex associations with CRC, with gender disparities noted. In terms of screening, non-invasive methods like fecal occult blood tests (FOBTs) are widely used, and combining biomarkers with iFOBT shows potential. Multi-omics techniques, including genomics and microbiomics, offer new avenues for CRC diagnosis. Overall, while evidence highlights the significance of inflammatory biomarkers in CRC risk prediction, larger prospective studies are urgently needed to clarify their roles due to existing inconsistencies and methodological limitations.

## Introduction

Among global malignancies, colorectal cancer (CRC) stands as one of the most frequently occurring, with the United States reporting approximately 140,000 newly diagnosed instances in 2021 (1). Furthermore, this form of cancer ranks as the third most significant contributor to cancer-induced fatalities, exhibiting a persistent upward trend in prevalence. The substantial occurrence rate of CRC poses a severe challenge to public health across diverse populations worldwide. Numerous factors are associated with the incidence rate of CRC, including genetic factors, lifestyle choices such as diet, smoking, alcohol consumption, lack of exercise, and alterations in the gut microbiot (2). The development of optimal biomarkers to predict the occurrence of CRC is necessary to identify populations at elevated risks of CRC. Therefore, the use of such biomarkers would facilitate proper interventions, such as changing lifestyles, removing risk factors, and routine screening, which could be made more specific for different populations.

Epidemiological research results indicate that chronic inflammatory states play a vital role in the onset and advancement of diverse malignancies, including CRC. The pathways connecting persistent inflammation to different cancer types have been extensively discussed in previous literature (3). Multiple investigations have indicated a correlation between systemic inflammatory indicators and CRC susceptibility, albeit with some conflicting outcomes. These indicators encompass acute-phase proteins such as C-reactive protein (CRP), cytokines like interleukin-6 (IL-6), and signaling molecules like tumor necrosis factor-alpha (TNF-α). A comprehensive analysis by Garcia-Anguita and colleagues in 2015 explored the interconnection between inflammatory markers and colorectal malignancy, emphasizing their potential as predictive tools for assessing CRC occurrence (4). Nevertheless, notwithstanding the extensive recognition of the significance of inflammatory cytokines in CRC, a multitude of unresolved enigmas still persist regarding the precise correlation between them and the incidence of CRC. For instance, the specific functions of diverse inflammatory cytokines during the pathogenesis of CRC, as well as their synergistic or antagonistic interrelationships, remain incompletely elucidated. The manner in which inflammatory cytokines interact with other tumor-associated signaling pathways to jointly modulate the progression of CRC also demands in-depth investigation. Additionally, the application value of inflammatory cytokines in the early warning, risk assessment, and prognosis determination of CRC awaits comprehensive evaluation (5).

While CRP’s rise in inflammation and general link to tumorigenesis are known, its connection to CRC risk is unclear due to inconsistent studies and gender/methodological differences. Besides CRP, mediators like IL-6, TNF - α, their receptors, and adipocyte-derived substances have complex, undefined roles in CRC development, with gender disparities adding more complexity. Traditional, simple FOBTs are widely used for population screening. However, combining biomarkers with advanced iFOBT shows promise for better diagnostic precision. Understanding these connections is essential as it could pave the way for the development of targeted preventive and therapeutic strategies, especially considering the influence of gender and methodological differences. This review aims to provide a holistic view of these screening and diagnostic developments, helping researchers and clinicians alike to make informed decisions and drive forward progress in the fight against CRC. In essence, it serves as a roadmap for future research and clinical practice, with the ultimate goal of improving patient outcomes.

## C-reactive protein

Serum C-reactive protein (CRP), a well-known inflammatory biomarker, exhibits an elevation in its concentration during the body’s inflammatory state. Inflammation has been established to have a profound and intricate connection with tumorigenesis. Chronic inflammation is capable of generating a tumor microenvironment that is highly favorable for the survival, and dissemination of tumor cells (6). In the presence of persistent inflammatory stimuli within the body, CRP levels escalate. Inflammatory cells, drawn to the affected sites, release cytokines such as ROS, TNF - α and IL-6 (7). These cytokines can promote angiogenesis within the tumor microenvironment, providing essential resources for tumor growth and facilitating metastasis (7–9). Essentially, the elevation of CRP due to inflammation sets the stage for tumorigenesis by creating a hospitable environment for cancer cells. However, chronically elevated CRP due to persistent inflammation can disrupt the normal functionality of immune cells. For example, CRP can impede the differentiation and effector functionalities of T cells, thereby diminishing the body’s anti-tumor immune response and creating a conducive environment for tumorigenesis (10, 11). High levels of CRP can modulate the equilibrium between cell proliferation and apoptosis through multiple signaling cascades. Within the cellular milieu, CRP can activate crucial pro-proliferative signaling pathways such as the PI3K/Akt pathway, which accelerates the cell cycle and promotes incessant cell division, heightening the risk of tumorigenesis (12). Conversely, the mechanisms that are typically responsible for inducing apoptosis under physiological conditions can be attenuated. For example, CRP binds activating Fcg receptors, activating PI3K/Akt, ERK, and NF-kB pathways, resulting in the survival and accumulation of abnormal cells that would otherwise undergo programmed cell death. These aberrant cells can then potentially progress to form tumors (13). Metabolic disorders like obesity and insulin resistance often go hand in hand with increased CRP. In obese individuals, adipose tissue secretes copious amounts of inflammatory mediators, which further drive CRP elevation. Additionally, these disorders alter the body’s metabolic state, for instance, causing high insulin levels. The combination of these metabolic changes and elevated CRP jointly promotes tumor development (14). Research has consistently demonstrated that there is a substantial modification in the constitution of gut microbiota in individuals with CRC compared to healthy controls (15). A healthy gut microbiota actively and protectively functions in sustaining the body’s immune homeostasis and modulating inflammation and can exert anti-cancer effects (16). However, once the balance of the gut microbiota is disrupted, leading to decline of beneficial bacteria and overgrow of pathogenic or opportunistic bacteria, trigered the activation of immune cells in the gut and subsequent inflammation, which may be manifested by alterations in CRP levels. As a result, it creates an inflammatory microenvironment that is conducive to the growth of tumor cells (17). Thus, by monitoring CRP, valuable insights can be gleaned regarding the status of gut microbiota and its potential association with CRC development. This approach may potentially act as a non-invasive and economically efficient supplement in the screening and monitoring of CRC.

A number of investigations have demonstrated that CRP is associated with the occurrence of cancer (18). A previous meta-analysis published in 2014, including 18 prospective studies, showed a 1.12-fold increment in CRC risk was correlated with one unit alteration in the natural log unit of CRP (19). However, this study was marred by significant heterogeneity, which stemmed from methodological and demographic differences among the included studies. All studies encompassed in this meta-analysis exhibited a positive connection between CRP and CRC risk, while the preponderance of studies was unable to detect a significant association between serum CRP levels and CRC risk (19). Subsequently, Another meta-analysis conducted two years subsequent refuted any association between circulating CRP levels and the risk of colorectal cancer CRC (20). It is also worth noting that several case-control studies included in this recent meta-analysis might have introduced biases in the circulating CRP levels, which might have been influenced by the disease process.

To build upon the meta-analysis conducted in 2014 (19). We conducted a literature review of prospective studies examining the correlation between highly sensitive CRP measurements and CRC occurrence from 2015 to 2023. **Table 1** provides a summary of the findings from investigations into the correlation between CRP and susceptibility to CRC.

**Table 1:** Studies of inflammatory biomarkers and colorectal cancer risk, published from 2015 to 2023.

Author, year	Country	Population	Study design	No. of cases	No. of controls/population	Inflammatory biomarkers	OR/HR (95% CI)
Koutroubakis, 2016 (35)	USA	Patients with ulcerative colitis	Prospective cohort	55	773	CRP	OR 1.33 (1.21–1.46)
Izano, 2016 (21)	USA	Health ABC	Prospective cohort	55	2490	CRP IL-6 TNF-alpha	HR 1.25 (0.60,2.59) HR 1.10 (0.52,2.36) HR 0.87 (0.45,1.70)
Murphy, 2015 (26)	USA	WHI-CT	NCC	401	802	CRP	OR 1.00 (0.69, 1.44)
Prizment, 2016 (23)	USA	ARIC	Prospective cohort	255	12300	CRP B2M	HR 2.21 (1.32, 3.70) HR 2.17 (1.39, 3.37)
Boden, 2020 (33)	Sweden	NSHDS and VIP	NCC	1010	1010	CRP	OR 1.04 (0.98, 1.10)
Wang, 2019 (25)	USA and Europe	GECCO	NCC and case-control	10 644	10 729	CRP-score	OR 1.07 (0.96, 1.20)
Wang, 2019 (25)	USA and Europe	CORECT	NCC and case-control	19 836	12 115	CRP-score	OR 1.02 (0.93, 1.12)
Nimptsch, 2015 (105)	Europe	EPIC	NCC	727	727	CRP-score	OR 1.74 (1.06, 2.85)
Ghuman, 2017 (31)	Sweden	AMORIS	Prospective cohort	4764	320 835	CRP Albumin Haptoglobin Leukocytes	HR 0.91 (0.77, 1.07) HR 0.02 (0.01, 0.03) HR 2.01 (1.86, 2.36) HR 1.322 (1.10, 1.56)
Song, 2016 (28)	USA	NHS	NCC	757	757	MIC-1 CRP IL-6 sTNFR-2	HR 1.52 (1.06, 2.18) HR 1.49 (1.09, 2.05) HR 1.23 (0.88, 1.71) HR 1.10 (0.78, 1.54)
Kim, 2016 (30)	USA	PHS	NCC	268	446	CRP IL-6 TNFR-2	OR 1.07 (0.69, 1.66) OR 1.46 (0.90, 2.35) OR 1.49 (0.94, 2.36)
Allin, 2016 (22)	Danish	CGPS	Prospective cohort	592	83397	CRP Fibrinogen Leukocyte count	HR 1.45 (1.17, 1.79) HR 1.97 (1.53, 2.52) HR 1.32 (1.07, 1.63)
Liu, 2022 (34)	China	Kailuan cohort	Prospective cohort	348	52 276	CRP	HR 1.13 (1.01, 1.23)
Dashti, 2022 (27)	USA	WHI-OS	NCC	456	839	CRP Leptin	OR 1.05 (0.93, 1.20) OR 1.09 (0.90, 1.33)
Kakourou, 2015 (47)	USA	CLUE II	NCC	173	345	IL-6	OR 2.09 (1.26, 3.46)
Marrone, 2021 (68)	USA	CLUE II	NCC	193	193	Adiponectin Leptin sTNFR2	OR 0.99 (0.43, 2.29) OR 0.81 (0.34, 1.90) OR 3.06 (1.19, 7.88)
Chandler, 2015 (81)	USA	WHS	NCC	275	275	Adiponectin	OR 0.86 (0.48, 1.56)
Inamura, 2016 (82)	USA	NHS	NCC	152	297	Adiponectin (lowset vs. highest)	OR 1.97 (0.82, 4.75)
Inamura, 2016 (82)	USA	HPFS	NCC	155	296	Adiponectin (lowset vs. highest)	OR 4.21 (1.52, 11.6)
Nost, 2021	UK	The UK Biobank	Prospective cohort	2401	439714	SII NLR PLR LMR	HR 1.47 (1.34, 1.61) HR 1.35 (1.22, 1.48) HR 1.36 (1.24, 1.5) HR 0.75 (0.67, 0.83)
Eichelmann, 2019	Europe	EPIC-Potsdam cohort	NCC	221	2329	chemerin	HR 1.81 (1.08, 3.05)
Kim, 2019 (48)	Korea	NCCK	case-control	696	1835	IL-6	OR 6.23 (4.10, 9.45)
Zhu, 2022 (24)	UK	The UK Biobank	Prospective cohort	34979	420964	CRP	HR 1.05 (1.02, 1.07)
Cilie, 2019	Netherlands	UCC-SMART cohort	Prospective cohort	107	7178	CRP	HR 1.05 (0.98, 1.12)
Bernard, 2021	Europe	EPIC-Heidelberg sub-cohort	Prospective cohort	3792	7767	CRP	HR 4.15 (2.55, 6.75)
Nimptsch, 2022 (105)	Europe	EPIC	Prospective cohort	822	1235	CRP	HR 0.92 (0.66, 1.28)

NCC, nested case-control study; OR, odds ratio; HR, hazards ratio; CRP, C-reactive protein; IL-6, interleukin-6; TNF-α, tumor necrosis factor-alpha; WHS, Women’s Health Study; Health ABC, Health, Aging, and Body Composition Study; WHI-CT, Women’s Health Initiative Clinical Trial; B2M, β-2 microglobulin; ARIC, the Atherosclerosis Risk in Communities Study; NSHDS, Northern Sweden Health and Disease Study; VIP, Vasterbotten Intervention Programme; GECCO, Genetics and Epidemiology of Colorectal Cancer Consortium; CORECT, Colorectal Transdisciplinary Study; AMORIS, Apolipoprotein MOrtality RISk study; NHS, Nurses’ Health Study; PHS, Physicians’ Health Study; CGPS, Copenhagen General Population Study; WHI-OS, Women’s Health Initiative Observational Study; EPIC, European Prospective Investigation into Cancer and Nutrition; NCCK, National Cancer Center - Korea HPFS, Health Professionals Follow-up Study; SII, systemic immune-inflammation index; NLR, neutrophil-to-lymphocyte ratio; PLR, platelet-to lymphocyte ratio; LMR, lymphocyte-to-monocyte ratio; UCC-SMART, Utrecht Cardiovascular Cohort - Second Manifestations of ARTerial disease.

It’s worth noting that prior longitudinal cohort studies have shown inconsistent results regarding this relationship (21). In a large-scale European cohort study involving hundreds of thousands of individuals, researchers evaluated baseline CRP levels through blood samples collected from participants and carried out a median follow-up period of 4.8 years. The results demonstrated a considerable increase in the risk of CRC among individuals with baseline CRP levels in the upper quartile, in contrast to those in the lower quartile. Notably, this correlation remained even after adjusting for conventional risk factors, such as age, gender, alcohol consumption, smoking history, and body mass index (BMI), with a hazard ratio (HR) of approximately 1.3-1.5 (22). Comparable studies in large U.S. cohorts and the UK Biobank cohort have likewise confirmed these findings (23, 24). Specifically, Long-term surveillance of community populations in specified regions indicated a favorable association between increased baseline CRP levels and the hazard of CRC., an association that was evident in both men and women, albeit slightly more pronounced in men (22).

A previous meta-analysis first highlighted the gender disparity (19). It revealed that elevated CRP was significantly associated with an increased CRC risk in men, yet no such link was found in women. To further investigate the female scenario, Wang et al. monitored 19,437 female participants from the Kailuan cohort for up to 5 years, quantifying their baseline serum CRP (25). Surprisingly, CRP levels did not correlate with CRC risk in this cohort. Another case-control study focused solely on postmenopausal women (26, 27), with no exogenous hormone use at baseline, also failed to establish a significant CRP-CRC association. However, a nested case-control study (28) with 84% postmenopausal women, 42% on menopausal hormone therapy, came close (p = 0.06) to showing a connection between CRP and CRC risk. Hormone replacement therapy has been demonstrated to decrease CRC occurrence in females (29). This suggests that endogenous estrogens likely protect postmenopausal women from CRC. In female CRC occurrence, CRP’s role appears subdued, potentially due to the complex hormonal environment overriding or interacting with CRP-mediated pathways. Interestingly, a nested case-control study in men with only 268 cases also had a negative result, perhaps because of the small sample size (30). Future research must consider gender stratification to unravel the complex mechanisms and develop more targeted CRC prevention and treatment strategies based on gender.

Most studies adopted high-sensitive CRP (hs-CRP), which enabled the discrimination of CRP concentrations below the level of 10 mg/L. However, one large-scale prospective study carried out in Sweden from 1985 to 1996 did not utilize hs-CRP during laboratory examinations (31). In the majority of studies, the average or median baseline CRP level ranged from 1 to 3 mg/L (25). In contrast, the mean CRP value in the Swedish cohort was 19.42 mg/L (31). Such a significant difference in the mean values indicates the limitations of previous CRP assays. Moreover, The lagging nature of the detection approach might potentially result in a lack of evidence that clearly demonstrates an association between CRP and CRC risk (31). The cut-off values also differed from each other. In most studies, CRP levels were divided into tertiles or quartiles. The lowest boundary varied between 1.0 and 1.3 mg/L, while the most commonly adopted highest boundary was 3 mg/L or higher. A unified cut-off value may provide consistency for subsequent work.

CRC predominantly affects the aged population. Approximately 90% of newly diagnosed CRC cases occur in populations exceeding 50 years of age, with a median age of diagnosis at 69 years (32). This age-related prevalence underlines the importance of timely intervention. The median enrollment age across recent studies ranges from 46 to 73 years, which can impact CRC incidence as it correlates with age. Follow-up times also differ, from 4.8 to 18 years, with a median time between blood sampling and diagnosis around 10 years. Crucially, studies like the European cohort in Denmark (22) found that the association between CRP level and CRC risk was most evident in the initial follow-up years. Prolonged detection intervals, as seen in a large-scale Swedish study (31) with an 18-year median follow-up, introduce confounding factors that obscure the CRP - CRC link. Boden et al.’s embedded case-control study (33) further explored this relationship. While overall pre-diagnostic CRP didn’t strongly correlate with CRC incidence, elevated CRP levels within five years prior to diagnosis were linked to advanced (stage III-IV) CRC. Given that chronic CRP elevation predisposes to CRC, optimizing CRP detection age, particularly focusing on those 50 and older, and shortening follow-up intervals, is essential. It would enhance CRP’s predictive power and enable more targeted CRC prevention strategies, leveraging the age-dependent nature of the disease.

A case-control study (33) showed that plasma CRP concentrations changed between two measurements over approximately 10 years in both CRC cases and controls, highlighting CRP’s dynamic nature. This implies that a single-time detection might not accurately reflect participants’ actual situation. Liu et al.’s prospective cohort study (34) on 52,276 subjects categorized them based on CRP-level trends. Those with an initial moderate CRP followed by an upward trend had a higher likelihood of developing CRC compared to those with consistently low levels. Interestingly, individuals with initially high CRP that later declined had a lower probability of CRC than those with stable low levels. Another observational cohort study (35) in ulcerative colitis (UC) patients (773 in total, with 50 developing CRC neoplasm during follow-up) found that five-year patterns of serum CRP were significantly associated with a higher CRC risk. A study on 55 CRC patients further contributed to understanding the CRP - CRC correlation (21). Moreover, lifestyle factors like non-visceral obesity, high-fat diet, smoking, and estrogen use were shown to influence the causal effect of CRP on CRC development (36). In conclusion, repeated CRP measurements during follow-up can better represent a person’s inflammatory status than a single one. As chronically elevated CRP levels play a vital role in CRC occurrence, dynamic CRP detection is essential for a more comprehensive assessment and prediction of CRC risk. In the future, it is expected that by optimizing the detection methods of C-reactive protein (CRP), unifying the detection cut-off values, focusing on optimizing the detection age for people aged 50 and above and shortening the follow-up intervals, and conducting stratified research fully considering gender differences, combined with multi-omics technologies to deeply explore its association with factors such as the gut microbiota, the risk of CRC can be accurately predicted, contributing to the formulation of personalized prevention and treatment strategies.

### IL-6

IL-6, being a significant mediator of inflammation, plays a critical role in chronic inflammatory disorders, including intestinal inflammatory diseases and autoimmune joint conditions (37). The mechanism of IL-6 in CRC is intricate. Firstly, in terms of promoting tumor cell proliferation, IL-6 binds to its receptor on cancer cells, activating the JAK/STAT3 signaling pathway (38). This leads to STAT3 phosphorylation and entry into the nucleus, upregulating genes like cyclin D1, driving cell cycle progression. Meanwhile, it modulates the PI3K/Akt pathway, facilitating cell survival and growth (39). Secondly, for inhibiting apoptosis, IL-6 activates STAT3 to enhance the expression of anti-apoptotic proteins like Bcl-2 and Bcl-xL, while concurrently repressing pro-apoptotic proteins such as caspase-3, thereby enabling tumor cells to evade apoptosis (40, 41). It also induces epithelial-mesenchymal transition (EMT), enhancing cell migratory and invasive capabilities (42). Finally, in the tumor microenvironment, IL-6 recruits immune cells like macrophages and neutrophils, releasing cytokines (43, 44). It also promotes regulatory T cells, suppressing the anti-tumor immune response, and overall fueling CRC development (45). Elevated IL-6 levels in blood serum have been detected across various malignancies, including those affecting the breast, colon, rectum, and lungs, indicating IL-6’s potential involvement in cancer progression (46). Since 2015, the literature has seen only several prospective studies examining the relationship between IL-6 and CRC susceptibility (21, 28, 30, 47). Among the published prospective studies on circulating IL-6, the majority of investigations did not identify a significant association with CRC risk. Nevertheless, merely a few studies indicated a significant connection between IL-6 and CRC risk (47). The CLUE II cohort study is particularly notable. It demonstrated a substantial association between IL-6 levels and the probability of CRC occurrence. In this cohort, those individuals having the highest plasma IL-6 concentrations exhibited approximately twice the risk of developing CRC in comparison to those with the lowest levels. Moreover, this association persisted to be significant even after adjusting for multiple factor such as educational background, smoking habits, body composition, utilization of aspirin or other anti-inflammatory drugs, diabetes management, and family history of CRC (47).

A meta-analysis involving 1,308 CRC cases from seven prospective studies was unsuccessful in manifesting a statistically substantial disparity between IL-6 and CRC risk (47). The strength of this result was limited by the small scale of all the included studies. The stratified analysis in the CLUE II cohort showed a stronger positive association between IL-6 and the incidence of colon cancer rather than rectal cancer. Similar results were also observed in the stratified meta-analysis. In A prospective study as part of the Health ABC project, which involved 2,490 individuals, subjects possessing IL-6 levels within the middle range exhibited a significantly heightened probability of developing colorectal malignancies in comparison to those with the lowest range (21). Interestingly, individuals with the highest IL-6 measurements were also associated with an increased incidence of CRC relative to the bottom quartile. The relatively small number of cases (15) in the uppermost tertile might explain this unexpected result. A study involving 2,531 subjects revealed that the plasma IL-6 concentrations in individuals diagnosed with CRC were notably higher compared to the control group. Individuals with IL-6 readings in the top 25% had a substantially higher probability of developing CRC than those in the bottom 25%. These findings suggest that IL-6 plasma levels could potentially serve as a preliminary indicator for the detection of CRC (48).

Some studies have reported that IL-6 levels are higher in neutropenic CRC patients with advanced clinical staging, suggesting that IL-6 may be closely related to the altered biological properties of colorectal tumor cells (49). Another study based on 164 pre-treatment Asian CRC patients also found that elevated IL-6 levels in patients may be associated with a higher risk of early cancer progression in CRC, indicating that IL-6 levels may act as an important predictor for CRC progression and poor prognosis (50, 51).

Most of the existing studies, especially those included in the meta-analysis, suffered from relatively small sample sizes. Thus restricting the reliability and generalizability of the results. CRC is a highly heterogeneous disease with significant differences in tumor biological characteristics among different stages, locations (colon versus rectum), and ethnic populations. Although some studies have conducted stratified analyses, they might still not have fully accounted for this complex disease heterogeneity when analyzing the association between IL-6 and CRC. This could potentially interfere with accurately determining the role of IL-6 in the occurrence, development, and prognosis of CRC. Future research could focus on expanding sample sizes, thoroughly analyzing the differences in the role of IL-6 in different stages, locations (colon versus rectum), and ethnic populations of colorectal cancer, integrating multi-dimensional factors, and precisely elucidating its complex mechanisms with the aid of advanced technologies, so as to accurately transform IL-6 plasma levels into reliable indicators for the early detection and prognostic judgment of colorectal cancer, facilitating the implementation of personalized medicine.

### TNF - α and sTNFR

TNF-α is a multifunctional cytokine produced mainly by immune cells such as macrophages and monocytes. It activates a series of signal transduction pathways by binding to specific receptors within cells. TNF - α has various biological functions, including regulating immune responses, cell proliferation, apoptosis, and inflammatory reactions. This cytokine contributes to inflammatory processes, tissue invasion, and cancer spread, exerting its effects through interaction with specific cell surface proteins, namely TNFR1 and TNFR2 (52). In the context of CRC, TNF - α exerts multiple effects. It promotes tumor cell proliferation by activating the NF-κB pathway (53). Simultaneously, TNF - α can induce apoptosis in cancer cells through the activation of the caspase cascade (54). Additionally, TNF - α plays a crucial role in the immune response against CRC. TNF - α plays a crucial role in the immune response against CRC. It activates immune cells, such as macrophages and T cells. These cells can recognize and attack cancer cells. TNF - α also promotes the recruitment of immune cells to the tumor site. However, in some cases, TNF - α can lead to immune evasion. For example, it may suppress the immune response by reducing the expression of major histocompatibility complex class II molecules on cancer cells (55, 56), and up-regulate PD-L1 expression (57). Furthermore, TNF - α is involved in the metastasis of CRC. It promotes tumor cell invasion and migration by regulating the extracellular matrix (58). At the molecular level, this phenomenon is intricately linked to TNF’s ability to activate STAT3-NF-κB signaling pathways, which enhance cancer cell growth and dissemination (59). Under the stimulation of TNF, chondroitin polymerizing factor 2 (CHPF2) undergoes phosphorylation at the T588 residue mediated by MEK. This phosphorylation event endows CHPF2 with the ability to interact with both TAK1 and IKKα. The consequently enhanced binding between TAK1 and IKKα triggers an increase in the phosphorylation of the IKK complex, subsequently activating the NF-κB signaling pathway. Activation of this pathway ultimately leads to the upregulation of the expression of early growth factors (EGR1), which plays a crucial role in promoting the proliferation and metastasis of CRC cells. This molecular mechanism provides valuable insights into the oncogenic processes underlying CRC and may serve as a potential target for future therapeutic interventions (59).

Clinical research has demonstrated markedly increased TNF - α concentrations in the blood of individuals with colorectal malignancies when compared to healthy subjects (60). Moreover, colorectal cancer patients exhibiting higher TNF - α levels typically experienced less favorable outcomes than those with lower concentrations (61, 62). Recently, some studies have been conducted to investigate the association between CRC risk, TNF - α, and TNFR2. The soluble form of TNFR2 (sTNFR2) serves as an indicator of TNFR2 upregulation during inflammatory states. Findings from CRC patients in the Nurses’ Health Study and the AGARIC multicenter case support the notion that elevated levels of sTNFR2 are significantly and positively associated with CRC risk (63, 64). In addition, higher sTNFR2 levels in CRC patients were associated with higher mortality, but not with CRC-specific mortality (64). The involvement of TNFR2 in CRC development and advancement suggests its potential as a therapeutic focus (65). TNFR2 were proposed as a novel hopeful tumor immune target in 2017, playing a complex role in tumor development (66). It drives tumor cell proliferation and is linked to the immunosuppressive function of Treg cells, contributing to an immunosuppressive tumor microenvironment.

While the longitudinal study of 3,075 individuals aged 70-79 showed a correlation between TNF - α serum concentrations and cancer susceptibility and an increased occurrence of colorectal malignancies in those with high TNF - α levels (67), two nested case-control investigations failed to establish significant links between TNFR2 and CRC likelihood (28, 30). This lack of consistency across different study designs makes it difficult to draw firm conclusions about the true relationship between these factors and CRC.

Additionally, the embedded case-control analysis had a gender-based variation in its findings, with statistical significance seen only among male subjects and no effect in females (68). This variation complicates the understanding of the role of the studied factors in CRC as it indicates that there may be additional variables (such as sex hormones) that need to be accounted for, and it’s not clear if the observed effects are generalizable across genders (69).

Concerns have focused on the effect of TNF-inhibitor (TNFi) on CRC, given that TNF - α is likely involved in the carcinogenesis of CRC. An unexpected conclusion was drawn by an early meta-analysis of randomized controlled trials, which reported that patients receiving higher doses of anti-TNF antibodies had a significantly higher incidence of malignancies compared to those receiving lower doses in rheumatoid arthritis treated with TNFi (70). On the contrary, three cohort studies conducted on inflammatory bowel disease (IBD) patients demonstrated that TNFi reduced the incidence of CRC (47, 71, 72). IBD is marked by prolonged inflammation, which results in a higher occurrence of CRC in comparison to the general population (73). This makes CRC a well-established sequela of long-standing IBD. Given the association between IBD and increased CRC risk, anti-inflammatory drugs used for IBD have been investigated for their impact on CRC development. Non-steroidal anti-inflammatory drugs (NSAIDs), commonly exemplified by aspirin, are widely used to treat inflammatory and cardiovascular problems and are recognized as chemoprevention agents. Their mechanisms chiefly involve inhibiting COX and prostaglandin E2 pathways, plus COX-independent ones. Systematic reviews and meta-analyses show aspirin use is linked to fewer colonic adenomas and CRCs, especially in younger to middle-aged groups. Strong evidence also supports the preventive power of both low- and high-dose aspirin in hereditary conditions like FAP and Lynch syndrome. This is due to their anti-inflammatory trait, ability to boost immune responses, trigger apoptosis, and halt angiogenesis, making them potentially beneficial in cancer prevention and treatment (74). TNF blockade promotes mucosal healing in patients with inflammatory bowel conditions, potentially mitigating abnormal cell growth and subsequent tumor formation in the colon while modifying microbiota composition and activities, thus attenuating colorectal carcinogenesis (75). Although dysregulated inflammation is the cause of both IBD and the development of colitis-associated CRC, the issue of whether treating the underlying inflammation can reduce the risk of CRC remains unclear in the current literature. A large-scale population study in the US, involving 62,007,510 patients, demonstrated that patients with IBD treated with anti-TNF agents had a lower rate of CRC. The observational study encompassing 6357 patients with IBD presents several constraints. The relatively brief mean follow-up duration of approximately 1 year implies that the long-term ramifications of TNF - α antagonist exposure on cancer risk might not be comprehensively apprehended (76). Long-term investigations are requisite to precisely evaluate the connection between TNF - α antagonist therapy and cancer risk in IBD patients. Recently, a Phase I trial of the TNF - α inhibitor certolizumab plus chemotherapy showed a relatively good median PFS period and effective rate in lung cancer patients (77). In preclinical studies, TNFR2 antibody therapy has shown effectiveness either alone or in combination with classical PD-1/CTLA-4 antibodies as it suppresses colorectal cancer and augments the efficacy of anti-PD1 immunotherapy by blocking TNF- α/TNFR2 signaling and decreasing CCR8+T regulatory cells (66, 78). Looking forward, with the dual roles of TNF - α and the potential of TNFR2 as a therapeutic target, further comprehensive and long-term studies are warranted to clarify their precise mechanisms in CRC, optimize related therapies, and ultimately improve the prognosis for colorectal cancer patients.

## Adiponectin and leptin

Adiponectin and leptin are endocrine factors secreted by adipocytes, and they influence the production of inflammatory mediators by adipocytes (79). It has been reported that adiponectin can suppress cancer development by inhibiting TNF-α. Additionally, recent investigations suggest that lipocalins may contribute to limiting neoplastic growth in colorectal malignancies by influencing diverse cellular signaling mechanisms (80). When it comes to the relationship between adiponectin levels and colorectal cancer incidence, a comprehensive review of 26 studies failed to find a significant link (79). However, further analysis showed an interesting gender difference. Elevated adiponectin concentrations were correlated with a reduced likelihood of CRC in male subjects, but this was not the case for females. We thoroughly examined prospective embedded case-control investigations (68, 81, 82), among them, one study demonstrated that decreased adiponectin levels were associated with a quadrupled incidence of CRC in men. In contrast, an embedded case-control analysis focusing on female participants did not find significant results regarding the impact of adiponectin on CRC susceptibility (81). The reasons behind this gender disparity remain elusive and require additional research. Recent findings from multiple studies have consistently shown that lower adiponectin concentrations correlate with heightened CRC susceptibility (83, 84). The differences in high-molecular-weight (HMW) adiponectin fractions and non-HMW adiponectin fractions have also been investigated. Many publications have demonstrated that HMW adiponectin fractions are involved in the process of insulin resistance, while non-HMW fractions play crucial roles in the inflammatory process. A meta-analysis including 7,554 CRC patients from 48 studies intended to investigate the difference in the HMW and non-HMW fractions of adiponectin on CRC risk (85). The result of this meta-analysis suggested that non-HMW adiponectin fractions significantly reduced the risk of CRC, while HMW adiponectin fractions did not. In summary, the role of adiponectin in CRC is complex, with gender differences and distinctions between different fractions influencing its association with the disease risk, and more research is needed to fully understand these aspects.

Leptin is an adipocytokine with pro-inflammatory properties, and increased secretion has been observed in obese states. As a lipoprotein enzyme, leptin is directly related to the proliferation and apoptosis process of tumor cells and promotes angiogenesis within tumor tissues through signaling pathways (86). Moreover, data suggests that leptin can play other roles in immune response, tumor infiltration and metastasis (87). Leptin expression shows inconsistency in normal colonic mucosa, colonic adenomas, and CRC (88). A large volume of literature reports that leptin plays a crucial role in the progression and pathogenesis of CRC. For example, Tutino et al. showed that high serum leptin levels are an independent risk factor (89). However, this finding is somewhat controversial. Several studies have shown a link between elevated serum leptin levels and increased CRC risk, but this conclusion is somewhat controversial (90, 91). Recently, two nested case-control studies showed that leptin was not associated with CRC risk (27, 68). In a similar vein, a meta-analysis conducted by Wang and colleagues concluded that the presence of leptin in the bloodstream did not significantly impact CRC susceptibility (79). There is a great diversity in sample sizes, body weights and age groups among these studies, which may lead to differences in the results.

## Early detection of colorectal cancer

Early diagnosis of CRC can enhance overall survival, population-based screening programs have been carried out in numerous countries globally (92). Full colonoscopy, which is widely recognized as the reference standard for CRC detection and is often employed in primary CRC screening, has significant disadvantages, including being invasive, expensive, and sometimes causing complications. Consequently, the two-step screening approach has emerged as the globally favored CRC screening strategy. This entails initial non-invasive testing, with secondary colonoscopies reserved for those who test positive (93). As non-invasive and low-cost screening methods, fecal occult blood tests (FOBTs) are extensively applied in CRC screening. The analysis included 597 participants, among whom 179 had CRC, 193 had advanced adenoma, and 225 were free from colorectal neoplasm. In CRC patients compared to participants without neoplasm, significant differences in blood levels of CRP, serum CD26 (sCD26), and tissue inhibitor of metalloproteinases 1 (TIMP-1) were observed. For CRC and advanced adenoma detection, single blood markers showed significantly lower sensitivities than FOBTs. However, the combination of inflammatory markers with iFOBT moderately increased the AUC for advanced adenomas, although only marginally from 0.683 (iFOBT alone) to 0.710 - 0.729 (with blood tests) (94). In a case-control study, the combinations of CEA + IL-8 also don’ t seem a viable alternative to FOBT-based CRC screening. Nevertheless, the potential of combining them with iFOBT merits further exploration (95).

Alexandre Loktionov’s team analyzed the biomarkers in colorectal mucus (CM) samples collected by a novel, non-invasive technique. The diagnostic sensitivity of hemoglobin attained 80.0% with a specificity of 94.3% in the ‘screening’ context, offering high sensitivity and specificity values for CRC detection. Significantly, in CM samples, increased concentrations of CRP, TIMP1, M2-PK, MMP9, and PADI4 could also serve as CRC biomarkers, but their efficacy was inferior to that of hemoglobin (96). Another case-control study was carried out to measure proteins in CM. Six biomarkers, namely hemoglobin, TIMP1, M2-PK, PADI4, CRP and MMP9 yielded high AUC values ranging from 0.857 to 0.943, along with promising combinations of sensitivity (70.6% - 88.2%) and specificity (77.1% - 94.3%) (97). Recent studies propose that combined serological biomarkers have potential in blood-based CRC screening research. Plasma CRP and soluble urokinase plasminogen activator receptor (suPAR), which indicates inflammatory activity, demonstrated significant correlations with newly diagnosed cancer during follow-up after adjusting for age and sex. The combination of CRP and suPAR yielded the highest AUC and sensitivity, with a negative predictive value reaching 93.4% (98). A mass spectrometry-based test of blood serum protein biomarkers for CRC detection showed a panel consisting of four proteins (CD44, GC, CRP, and ITIH3) showed the highest efficacy in differentiating regional from localized cancers, yet not in predicting CRC occurrence compared to cancer-free individuals (99).

A large multicenter prospective study on symptomatic patients got promising results for CRC detection via biomarker panels. Combining 8 biomarkers improved early CRC and adenoma detection (AUCs 0.76 - 0.84). A reduced model with 4 key biomarkers (CEA, CyFra21-1, Ferritin, and hs-CRP), along with age and gender, had similar AUCs as the full one (100). In the screening population with hemoglobin higher than 100 ng/mL, a predefined algorithm based on clinically available biomarkers, namely FIT, age, CEA, hsCRP and Ferritin, shows better diagnostic efficacy for CRC compared with using FIT alone (101). In the context of the article, it seems that the combination of multiple factors like CEA, CRP, Ferritin, age, and the use of iFOBT can potentially lead to a more accurate and effective screening approach for CRC.

### Multi-omics techniques

Advanced high throughput “multi-omics” techniques, namely genomics, transcriptomics, proteomics, microbiomics as well as metabolomics, offer minimally invasive or non-invasive means for the diagnosis of CRC, with each having its own strengths and weaknesses. Both transcriptomics and proteomics are closely related to the physiological state of organisms and thus possess great potential in treatment. Metabolomics can rapidly and precisely characterize the phenotypes of organisms and their metabolic pathways. It can directly detect changes in metabolites within organisms, which reflect the physiological and pathological states more intuitively.

### Genomics

Genomics shows high efficiency in evaluating CRC susceptibility and genetic risks by analyzing genomic sequences to identify relevant gene mutations and genetic markers. Single nucleotide polymorphisms (SNPs) constitute the most common form of genetic variation in humans. It has been noted that the genetic polymorphisms of CRP can reflect plasma CRP levels (102). Notably, CRP-related SNPs have the potential to interfere with the binding of transcription factors to the CRP gene promoter region (103) and influence the synthesis and secretion of CRP into the bloodstream. Several SNPs in the CRP gene have been shown to be associated with different CRP levels. For instance, The minor alleles of rs1130864 and rs3093059 were associated with increased CRP levels (both P < 0.001). Conversely, the minor alleles of rs1205, rs1800947, and rs2246469 were associated with decreased CRP levels (all P < 0.001) (104). The association between CRP-related SNPs and the occurrence of CRC is complex and not yet fully understood. Some studies have attempted to explore this relationship, but the results have been inconsistent. Specifically, several investigations found a statistically significant correlation between CRP concentrations based on single nucleotide polymorphisms (SNPs) in the CRP gene and CRC risk (105, 106). However, the statistical power of the conclusions drawn from these studies was limited due to relatively small sample sizes. Large-scale studies like the Mendelian randomization analysis involving a large number of CRC cases and controls found no significant association between genetically elevated CRP concentration (as influenced by certain SNPs) and CRC risk (25). A pooled analysis found that there was no significant positive correlation between these two variants and cancer incidence (107). However, stratification analysis indicated that the CRP rs1205 C>T polymorphism was correlated with an increased risk of cancer in Asians, but not in European populations, suggesting that the relationship between CRP-related SNPs and CRC susceptibility may vary by ethnicity. A meta-analysis incorporating 80361 cases and 78712 controls from 97 case-control studies was carried out to evaluate the association between IL-6 promoter polymorphisms and cancer susceptibility (108). Pooled analysis revealed that IL-6 promoters rs1800795 and rs1800796 were significantly correlated with an augmented risk of cancer in Asia and Caucasian. However, rs1800797 was significantly associated with a higher risk of cancer in Caucasian, but not in Asia, indicating a disparity between ethnic groups. A number of previous studies have proven that TNF-α-308G/A polymorphism was associated with the susceptibility of CRC. However, the findings are still debatable among the populations (109–112). The conflicting results are perhaps due to the small sample size in various studies, the defection of case-control study design and ethnicity differences. Future large prospective studies are warranted to identify the association between SNPs and the risk of CRC.

### Microbiomics

The gut microbiota has emerged as a significant player in human health and disease, with mounting evidence suggesting its profound influence on CRC development (113). Microbiomics plays a significant role in understanding the risk factors and potential screening methods for CRC. A case-control study observed significant positive correlations between specific bacterial species, namely Fusobacterium nucleatum, Parvimonas micra, and Porphyromonas, and two key inflammatory markers: hs-CRP and IL-6. These findings suggest a potential mechanistic link between the presence and abundance of these bacteria and the inflammatory response. Abnormal microbiota composition has been strongly implicated as an etiologic factor in CRC. With advanced sequencing technologies like metagenome sequencing and pyrosequencing, numerous bacteria have been identified to correlate positively with CRC incidence (114–116). For instance, Fusobacterium, F. nucleatum are significantly elevated in CRC patients compared to healthy controls (114). This bacterium’s ability to interact with the host immune system underlines its importance in CRC pathogenesis. Alterations in the intestinal barrier allow microbes to trigger local inflammation, accompanied by the upregulation of inflammatory factors like IL-17, Cxcl2, Tnf-α, and IL-1, which in turn facilitate polyp and cancer progression in mice (117). When integrated with known clinical risk factors of CRC, data from the gut microbiome notably augment the capacity to discriminate between healthy, adenoma, and carcinoma clinical groups in comparison to the sole utilization of risk factors. In a specific study, the combination of bacterial markers with fecal immunochemical test (FIT) achieved remarkable outcomes. The sensitivity of Fn detection escalated from 82.0% to 92.8%, and that of a four-bacteria panel also elevated from 83.8% to 92.8%, accompanied by enhanced positive predictive value and negative predictive value (118).

## Prospects for the future

The identification of individuals with a high future risk of developing CRC and those with negligible risk is essential for the development of personalized screening strategies. For example, high-risk individuals can be screened more frequently (such as annual FIT instead of biennial) or start screening earlier, while low-risk individuals can have less frequent screening. This approach provides optimal prevention for high-risk individuals and safeguards those with negligible risk from potential screening-related adverse effects. Longitudinal risk models are established to identify these distinct groups.

In the field of CRC prevention and control, accurately determining those at an elevated risk of developing CRC and those with minimal risk is essential for creating personalized screening plans. Cohort studies related to CRC have provided valuable insights into this area. Numerous large-scale cohort studies have consistently shown that multiple factors contribute to CRC risk stratification. Age is a significant factor; typically, the incidence of CRC starts to increase significantly after the age of 50. However, genetic predisposition can be more important in some cases. For example, individuals with a family history of CRC, especially those with affected first-degree relatives, have a higher risk. Lynch syndrome, a hereditary condition, greatly increases the likelihood of CRC development. For high-risk groups, screening should not only start earlier, perhaps a decade or more before the general recommended age, but also be more frequent. Additionally, more invasive and highly sensitive procedures like colonoscopy may need to be included at regular intervals to detect early-stage lesions more accurately.

Longitudinal risk models are powerful tools in this context. These models integrate various variables such as demographic data, genetic profiles, lifestyle choices, and medical histories. By using advanced statistical techniques and machine learning algorithms, they continuously monitor and update an individual’s CRC risk over time. For example, in a long-term cohort study spanning several decades, participants’ data on diet, exercise, disease diagnoses, and genetic test results were collected periodically. Every 3-5 years, the risk model recalibrated each participant’s risk level. Those whose risk increased were promptly adjusted to more intensive screening protocols, while those with stable or decreasing risks maintained or adjusted their screening regimens accordingly. This dynamic approach ensures the optimal allocation of healthcare resources, providing maximum preventive benefits to high-risk individuals and protecting low-risk individuals from unnecessary screening - related harms, thus revolutionizing CRC screening and prevention.

## Conclusion

Inflammatory biomarkers, including CRP, IL-6, TNF - α and its receptors, adiponectin and leptin, play complex roles in colorectal cancer development. Although their associations with CRC risk have been investigated, inconsistent results and gender differences remain, highlighting the need for further large-scale prospective studies. Non-invasive screening methods, such as FOBTs and combinations of biomarkers with advanced iFOBT, show potential for CRC detection. Multi-omics techniques, especially genomics and microbiomics, offer new avenues for CRC diagnosis and understanding its underlying mechanisms, but each has its own limitations. Future research should focus on optimizing detection methods, unifying cut-off values, considering gender stratification, shortening follow-up intervals, and integrating multi-omics data to accurately predict CRC risk. Longitudinal risk models can help develop personalized screening and prevention strategies, which are crucial for improving patient outcomes in the fight against CRC.
